# Supplementary material for: Monovision-induced motion illusions in presbyopic and non-presbyopic populations
Source: bioRxiv. 2025 Oct 22:2025.10.21.683743. Preprint. [Version 1] doi: 10.1101/2025.10.21.683743 (PMC12633227; doi:10.1101/2025.10.21.683743)
Supplement: 1 [file NIHPP2025.10.21.683743V1-supplement-1.pdf]

bioRxiv preprint doi: https://doi.org/10.1101/2025.10.21.683743; this version posted October 22, 2025. The copyright holder for this preprint (which was not certified by peer review) is the author/funder, who has granted bioRxiv a license to display the preprint in perpetuity. It is made available under aCC-BY 4.0 International license.

Supplement: Monovision-induced motion illusions in the presbyopic and general populations

Victor Rodriguez-Lopez[1,2,†]
Callista M. Dyer[3,†]
Johannes Burge[3,4,5]

[1.]Institute of Optics, Spanish National Research Council, IO-CSIC, Madrid, Spain
[2.]Department of Psychology, University of Pennsylvania, Goddard 426, 3710 Hamilton Walk, Pennsylvania PA 19104
[3.]Neuroscience Graduate Group, University of Pennsylvania, Goddard 426, 3710 Hamilton Walk, Pennsylvania PA 19104
[4.]Bioengineering Graduate Group, University of Pennsylvania, Goddard 426, 3710 Hamilton Walk, Pennsylvania PA 19104
[5.]Lead contact
[†.]Co-first authors

bioRxiv preprint doi: https://doi.org/10.1101/2025.10.21.683743; this version posted October 22, 2025. The copyright holder for this preprint (which was not certified by peer review) is the author/funder, who has granted bioRxiv a license to display the preprint in perpetuity. It is made available under aCC-BY 4.0 International license.

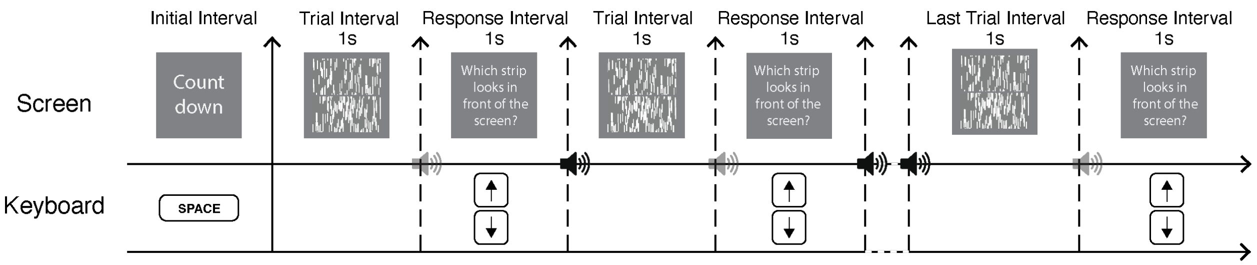

Figure S1. Temporal sequence of trials in a block of data collection. Each block began with a countdown. At the end of the countdown, the experimenter pressed the space bar to synchronize the stimulus video with data collection. On each trial, the target stimulus was shown, after which the participant responded with a key press to indicate whether the top or bottom strip appeared to be in front. The stimulus interval and the response interval lasted for one second. The onset of the response interval was marked by a sound. After the response interval ended, another sound indicated whether the subject's response was on time (high-pitch sound) or late (low-pitch sound). Late responses were discarded, but were also exceedingly rare; less than one percent of trials.

bioRxiv preprint doi: https://doi.org/10.1101/2025.10.21.683743; this version posted October 22, 2025. The copyright holder for this preprint (which was not certified by peer review) is the author/funder, who has granted bioRxiv a license to display the preprint in perpetuity. It is made available under aCC-BY 4.0 International license.

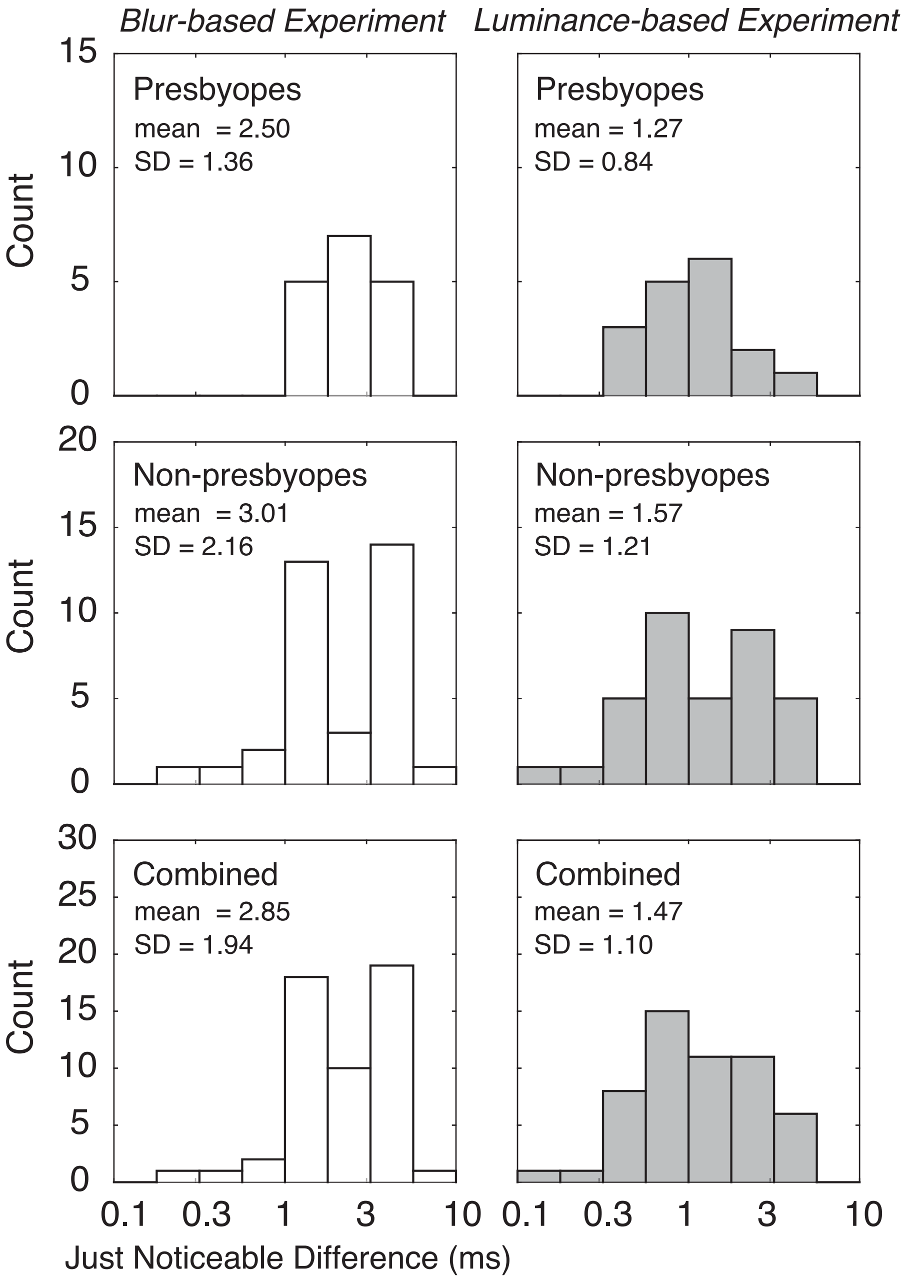

Figure S2. Just noticeable differences (JNDs) for presbyopic, non-presbyopic, and combined (or general) populations (top, middle, and bottom rows, respectively) in the blur- and luminance-based experiments (left and right columns, respectively). The detection threshold is specified by the JND.

bioRxiv preprint doi: https://doi.org/10.1101/2025.10.21.683743; this version posted October 22, 2025. The copyright holder for this preprint (which was not certified by peer review) is the author/funder, who has granted bioRxiv a license to display the preprint in perpetuity. It is made available under aCC-BY 4.0 International license.

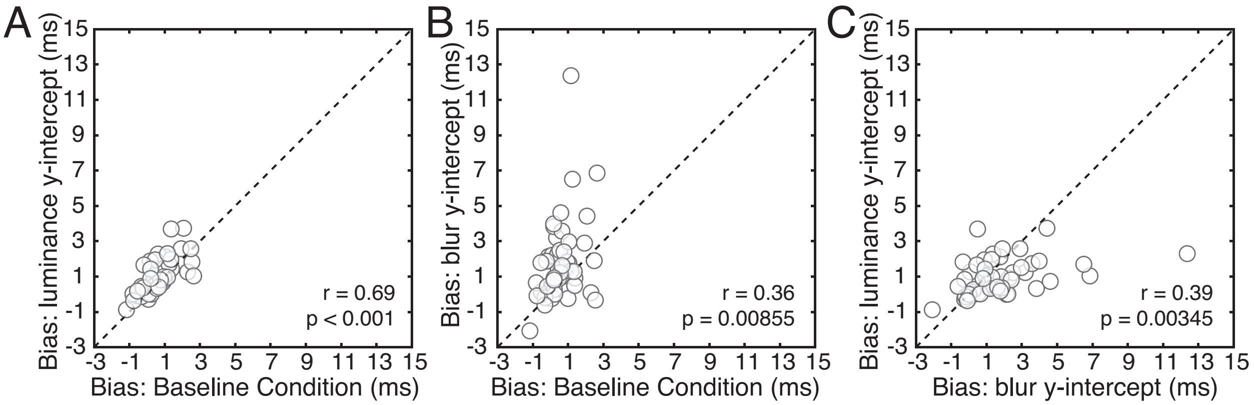

Figure S3. Biases across both populations (n=53). Bias was defined as the critical onscreen delay when no perturbation was applied to either eye. Biases were estimated in three different ways (see accompanying text). **A** Biases estimated from the baseline condition plotted against biases estimated from the y-intercept in the luminance-based experiment. **B** Biases estimated from the baseline condition plotted against biases estimated from the y-intercept in the blur-based experiment. **C** Biases in the blur-based experiment plotted against biases in the luminance-based experiment.

We measured bias—the critical onscreen delay when neither eye is perturbed—for each participant in three different ways: i) we collected data without perturbing either eye—a 'baseline' condition—and estimated the PSE using the analysis methods described above, ii) we took the y-intercept of the data connecting the PSEs in the luminance-based experiment (e.g. Fig. 2CF, sharp lines), and iii) we took the y-intercept of the line connecting the PSEs in the blur-based experiment (e.g. Fig. 2CF, blurry lines). Then, we scatter-plotted the bias estimates against one another. All three estimates of bias are correlated with one another. The blur-based estimate, however, is correlated less well than the other two, suggesting that optical blur may have somewhat asymmetrical effects across the two eyes, perhaps due to higher-order aberrations. The unique optics of each eye, and how it influences these effects, is a topic of potential interest to be explored in future work.
